# Preventive service utilization among low-income cancer survivors

**DOI:** 10.1007/s11764-021-01095-7

**Published:** 2021-08-19

**Authors:** Brenna E. Blackburn, Miguel Marino, Teresa Schmidt, John Heintzman, Brigit Hatch, Jennifer DeVoe, Laura Moreno, Nathalie Huguet

**Affiliations:** 1 Department of Family Medicine, Oregon Health & Science University, 3181 Sam Jackson Park Road, Portland, OR, USA; 2 Department of Family and Preventive Medicine, School of Medicine, University of Utah, 375 Chipeta Way, Salt Lake City, UT 84108, USA; 3 Division of Biostatistics, School of Public Health, Oregon Health & Science University – Portland State University, 3181 SW Sam Jackson Park Road, Portland, OR, USA; 4 OCHIN, Inc., 1881 SW Naito Pkwy, Portland, OR, USA

**Keywords:** Preventive service utilization, Low-income cancer survivors, Community health centers

## Abstract

**Purpose:**

Adequate access to and utilization of preventive services are vital among cancer survivors. This study examined preventive service utilization of cancer survivors compared to matched patients with no history of cancer among patients seeking care at community health centers (CHCs).

**Methods:**

We utilized electronic health record data from the OCHIN network between 2014 and 2017. Cancer survivors (*N* = 20,538) ages ≥ 18 years were propensity score matched to three individuals with no history of cancer (*N* = 61,617) by age, sex, region, urban/rural, ethnicity, race, BMI, and Charlson Comorbidity Index. Preventive screenings included cancer, mental health and substance abuse, cardiovascular, and infectious disease screenings, and vaccinations. Patient-level preventive service indices were calculated for each screening as the total person-time covered divided by the total person-time eligible. Preventive service rate ratios comparing cancer survivors to patients with no history of cancer were estimated using negative binomial regression.

**Results:**

Cancer survivors had higher overall preventive service utilization (incidence rate ratio = 1.11, 95% confidence interval = 1.09–1.13) and higher rates of cancer screenings (IRR = 1.16, 95% CI = 1.12–1.20). There was no difference between the two groups in mental health screenings.

**Conclusions:**

Cancer survivors were more likely to be up-to-date with preventive care than their matched counterparts. However, mental health and substance abuse screenings were low in both groups, despite reports of increased mental health conditions among cancer survivors.

**Implications for Cancer Survivors:**

With the growing number of cancer survivors in the USA, efforts are needed to ensure their access to and utilization of preventive services, especially related to behavioral and mental healthcare.

## Introduction

There are currently nearly 17 million cancer survivors in the USA, with that number expected to increase to over 22 million by 2030 [1]. The majority of these cancer survivors (67%) have survived more than 5 years. As cancer survivors are at an increased risk for many of the chronic conditions, it is vital that survivors receive appropriate preventive care. Preventive services can save lives and decrease healthcare costs by identifying illnesses earlier, managing them more effectively, and treating them before they develop into complicated, debilitating conditions [2, 3]. However, the focus of cancer survivors is likely on receiving cancer care over appropriate non-cancer-related preventive services such as diabetes and cardiovascular disease screenings. Studies examining preventive service use of cancer survivors show mixed results [4–15]. Cancer survivors have been observed to be more likely to have cancer screenings (e.g., mammograms, colorectal cancer screening, prostate-specific antigen test), but less likely to have non-cancer preventive screenings (e.g., influenza vaccination, cholesterol screening) [4–6].

Differences have been found with access to preventive services for cancer survivors ages 18–64 with private health insurance when compared to those with public insurance or no insurance [16]. Cancer survivors who are not able to continue working full-time may become eligible for Medicaid during cancer treatment due to lost wages, but may not be able to maintain Medicaid coverage after cancer treatment [17]. There has been no study on preventive service utilization among cancer survivors seen in community health centers (CHCs), which predominantly serve publicly insured or uninsured patients. Most previous studies focused on older populations using SEER Medicare data and/or only examining a specific cancer.

The aim of this study was to compare receipt of 14 cancer and non-cancer preventive services between cancer survivors and matched patients with no history of cancer in a network of CHCs across 14 states. We hypothesized that cancer survivors would have higher receipt of cancer screenings and similar receipt of non-cancer screenings than patients with no history of cancer.

## Methods

We utilized electronic health record (EHR) data from the OCHIN practice-based research network. All clinics within OCHIN use the same instance of Epic® EHR, which is centrally housed and managed at OCHIN. There are more than 350 OCHIN clinics located in 14 states across the USA with more than 2 million patients. We studied preventive service utilization between January 1, 2014, and December 31, 2017.

Patients were eligible for the study if they had an ambulatory visit within the OCHIN network in 2 years prior to the study period (2012–2013) to ensure they were established patients within OCHIN. Patients had to be 18 or older at the beginning of the study period (January 1, 2014) and had to have an ambulatory visit within the study period. Cancer survivors were included if they were diagnosed with cancer before the start of the study period with a malignant cancer. Cancer survivors were identified through diagnosis codes and problem lists within their medical records. Patients that were not alive at the end of 2017 were excluded.

### Preventive services outcome measures

The primary outcomes of the study were preventive screening indices. Preventive screening indices were calculated as rates where the numerator was the total person-time covered and the denominator was total person-time eligible [18]. This calculation results in a percentage of time “covered” by a preventive service (e.g., a mammogram “covers” an individual for breast cancer screening for 2 years). Therefore, by definition, each index was a number between 0 and 1: 0 being never received screenings and 1 being always covered (or on-time). Eligibility for each screening was determined using A or B recommendations from the U.S. Preventive Service Task Force. Supplemental Table 1 shows all the criteria used for each preventive service [18]. Individual preventive service indices were constructed for cancer screenings (breast, cervical, colorectal cancer screenings, and HPV vaccine), mental health and substance abuse screenings, cardiovascular screenings (blood pressure, HbA1c, and lipid screenings), and infectious disease screening and vaccination (chlamydia and hepatitis C screening, flu, and pneumonia vaccines). Additionally, an overall preventive service index was estimated averaging across all services combined to provide a comprehensive measure of overall preventive utilization [18].

### Statistical analysis

Each cancer survivor was propensity score matched with replacement to three patients with no history of cancer on age at the beginning of the study period, sex, urban/rural, ethnicity, race, Charlson Comorbidity Index (CCI), and body mass index (BMI). For the sub-analyses with specific cancer survivors (breast, prostate, colorectal, and lung), the cancer survivors were re-matched with propensity scores to ensure sex and age eligibility were appropriately matched upon.

For each preventive service index, we compared average receipt between cancer survivors and patients with no history of cancer and evaluated differences using standardized mean differences (SMD). The two groups are significantly different when the absolute value of the standardized mean differences is larger than 0.1. Given that some differences in patient characteristics between those with and without cancer were observed, and to account for overdispersion of patients with 0 as their preventive service index score, we utilized mixed effects negative binomial regression to estimate preventive service rate ratios comparing cancer survivors to patients with no history of cancer. Models utilized clinic random intercepts to account for clustering of patients within clinics. Models were run for each preventive service index for all cancer survivors combined compared to individuals with no history of cancer. Models were also run for each group of individual cancer survivors: breast, prostate, colorectal, and lung. All models controlled for age, insurance status, smoking status, body mass index, race, ethnicity, urban/rural, and number of visits during the study period [19]. These adjustments account for residual imbalance between the groups after propensity score matching. All analyses were completed in SAS 9.4.

## Results

There were 21,538 cancer survivors in the study population matched to 61,617 patients with no history of cancer. Cancer survivors were more likely to be male and non-White, have insurance, and be a former smoker (Table 1). Comparisons between breast, prostate, colorectal, and lung cancer survivors and their propensity score matched patients with no history of cancer are shown in Supplemental Tables 2 and 3. Across all cancers, cancer survivors had more visits on average than cancer-free individuals (mean = 13.1, vs mean = 8.1, SMD = 0.3).

Cancer survivors had a higher overall preventive service index than individuals with no history of cancer (mean = 38.4 vs mean = 34.4, SMD = 0.16), as seen in Table 2. Of the combined indices, all had an absolute value SMD of at least 0.1 with cancer screenings having the largest gap between cancer survivors and individuals with no history of cancer (SMD = 0.19).

Cancer survivors had a significantly higher overall preventive index score (incidence rate ratio = 1.11, 95% confidence interval = 1.09–1.13; Table 3). Cancer survivors had a 16% increased rate of cancer screenings compared to individuals with no history of cancer, with a 22% increased rate of colorectal cancer screenings. While cancer survivors appear to receive increased cancer, cardiovascular, and infectious disease preventive services, there was no significant difference for mental health and substance abuse index scores between the two groups (IRR = 1.02, 95%CI = 0.99–1.05).

Of the four individual cancer subgroups, breast cancer survivors had the highest IRR for overall preventive index score (IRR = 1.22, 95%CI = 1.16–1.27; Table 3). Prostate cancer survivors also had a significant overall preventive index score (IRR = 1.11, 95%CI = 1.03–1.17). Colorectal cancer survivors also had higher cancer preventive index score (IRR = 1.30, 95%CI = 1.15–1.48) and colorectal cancer surveillance ratio (IRR = 1.39, 95%CI = 1.19–1.63). Lung cancer survivors had a significantly lower rate for the overall preventive index (IRR = 0.80, 95%CI = 0.71–0.90). This decreased rate was also observed in the cardiovascular preventive index score (IRR = 0.85, 95%CI = 0.79–0.92) and the mental health and substance abuse index score (IRR = 0.75, 95%CI = 0.64–0.88).

## Discussion

We examined the preventive service utilization of cancer survivors and patients with no history of cancer in CHCs. Overall, cancer survivors had better rates of preventive service utilization when compared to individuals with no history of cancer, except for mental health and substance abuse services. Additionally, differences by cancer types were observed. Breast and prostate cancer survivors generally had better preventive services scores than patients with no history of cancer, while colorectal cancer survivors had similar rates of preventive services, and lung cancer survivors had significantly poorer preventive services rates. In addition, breast and colorectal cancer survivors are recommended to receive mammograms and colonoscopies, respectively, at increased frequency for surveillance and this likely impacted their increased preventive service indexes for cancer screenings. Breast cancer survivors also had increased cervical and colorectal cancer screenings when compared to those with no history of cancer; however, colorectal cancer survivors did not have increased breast and cervical cancer screenings.

Rates of receipt of mental health and substance abuse preventive services did not differ between cancer survivor and patients with no history of cancer. Cancer survivors have been observed to have increased rates of anxiety, depression, and suicide risk, including being more than twice as likely to have disabling psychological problems as adults without a previous cancer diagnosis [20–25]. Overall, less than a quarter of cancer survivors were screened for depression or substance abuse. These screenings were also low for the patients with no history of cancer. Low prevalence of mental and behavioral screenings is worrisome as mental disorders are associated with increased risk of a wide range of physical conditions such as heart disease and diabetes [26]. This is especially imperative as cancer survivors are already at an increased risk for these conditions [27, 28]. Mixed methods research is needed to understand the provider- and patient-related barriers to screening for mental health substance abuse.

Lung cancer has the lowest 5-year survival rate (19%) of any of the cancers, while breast and prostate have 5-year survival rates over 90% [1]. The poor survival rate likely explains why lung cancer survivors had significantly lower rates of preventive service utilization compared to those with no history of cancer. For these patients, the focus is likely on cancer survival over receiving screenings for other health conditions.

Our study has several limitations. First, the use of medical records may have led to an underestimation of preventive service utilization. Although the completeness of OCHIN’s preventive service utilization records has been previously validated [29], patients may have received care elsewhere and these records may not always be available in the CHC medical records. In addition, we may have patients who are lost to follow-up which is unaccounted for in this analysis. However, evidence suggests that 80% of established CHC patients, especially with chronic conditions, return for at least one visit in a 3-year period [30]. Second, due to identifying cancer survivors with diagnosis codes and problem lists and only moderate agreement between CHC EHRs and cancer registries, dates of cancer diagnosis were not always available and some cancer survivors were likely not identified [31].

This study also has several strengths. First, the study population comes from 14 states across the USA, which allows for generalizability among CHC patients. Second, this study has over 20,000 cancer survivors, which allowed us not only to study cancer survivors in general but also to have enough power to examine four specific groups of cancer survivors. Third, we were also able to study many different preventive services to get a clearer picture of what types of preventive services cancer survivors are utilizing and where improvement is needed.

In conclusion, cancer survivors overall are more up-to-date with preventive services than individuals with no history of cancer, especially for breast and prostate cancer survivors, but lack screening for mental health and substance abuse. Future directions should focus on understanding the facilitators and barriers to preventive services and on developing interventions to promote and facilitate all preventive services use and delivery in CHCs settings.

## Supplementary Material

Supplemental Table 3

Supplemental Table 2

Supplemental Table 1

## Figures and Tables

**Table 1:** Demographic characteristics of the cancer survivors and propensity score matched individuals with no history of cancer

	Cancer survivors	Individuals with no history of cancer
	*N* = 20,538	*N* = 61,617
Age		
18–19	37 (0.2)	104 (0.2)
20–34	975 (4.8)	2942 (4.8)
35–49	3003 (14.6)	9030 (14.7)
50–64	8589 (41.8)	25,777 (41.8)
65–79	6318 (30.8)	18,681 (30.3)
80 +	1616 (7.9)	5086 (8.3)
Sex		
Female	12,491 (60.8)	38,485 (62.5)
Male	8047 (39.2)	23,132 (37.5)
Ethnicity		
Hispanic	2110 (10.3)	6463 (10.5)
Non-Hispanic	17,622 (85.8)	52,930 (85.9)
Missing	806 (3.9)	2223 (3.6)
Race		
White	17,237 (83.9)	52,070 (84.5)
Black or African American	1966 (9.6)	5988 (9.7)
Asian	428 (2.1)	1237 (2.0)
American Indian or Alaska Native	138 (0.7)	294 (0.5)
Native Hawaiian or other Pacific Islander	44 (0.2)	83 (0.1)
Multiple races	111 (0.5)	227 (0.4)
Missing	614 (3.0)	1718 (2.8)
Federal Poverty Level, patient level		
< 138%	8336 (40.6)	25,185 (40.9)
138–200%	1516 (7.4)	4688 (7.6)
> 200%	1906 (9.3)	5560 (9.0)
Missing	8870 (42.8)	26,184 (42.5)
Insurance status at most recent visit		
Private	3297 (16.1)	9794 (15.9)
Medicaid	43,01 (20.9)	10,979 (17.8)
Medicare	8318 (40.5)	22,664 (36.8)
Uninsured	4132 (20.1)	16,363 (26.6)
Other/unknown	490 (2.4)	1817 (3.0)
Patient region of residence		
West	17,075 (83.1)	49,136 (79.7)
Southwest	79 (0.4)	510 (0.8)
Midwest	1953 (9.5)	7719 (12.5)
Southeast	1278 (6.2)	3767 (6.1)
Northeast	62 (0.3)	280 (0.5)
Missing	91 (0.4)	205 (0.3)
Body mass index		
Underweight (< 18.5 kg/m^2^)	348 (1.7)	881 (1.4)
Normal (18.5–24.9 kg/m^2^)	5125 (25.0)	15,309 (24.9)
Overweight (25.0–29.9 kg/m^2^)	6102 (29.7)	18,252 (29.6)
Obese (≥ 30 kg/m^2^)	7504 (36.5)	22,619 (36.7)
Missing	1459 (7.1)	4556 (7.4)
Smoking status		
Current smoker	4605 (22.4)	14,787 (24.0)
Former smoker	6238 (30.4)	16,144 (26.2)
Never smoker	8900 (43.3)	27,268 (44.3)
Missing	795 (3.9)	3418 (5.6)
Rurality		
Rural	7195 (35.0)	21,236 (34.5)
Urban	13,254 (64.5)	40,176 (65.2)
Missing	89 (0.4)	205 (0.3)
Charlson Comorbidity Index		
0	5519 (26.9)	16,855 (27.4)
1	4498 (21.9)	13,271 (21.5)
2 +	10,521 (51.2)	31,491 (51.1)
Number of visits between 2014–2017		
0	4722 (23.0)	17,197 (27.9)
1–4	3117 (15.2)	10,363 (16.8)
5–12	5113 (24.9)	15,099 (24.5)
13 +	7586 (36.9)	18,958 (30.8)

Cancer survivors were included if they were diagnosed with malignant cancer before the start of the study period. Cancer survivors were identified through diagnosis codes and problem lists within their medical records. Each cancer survivor was propensity score matched to three patients with no history of cancer on age at the beginning of the study period, sex, urban/rural, ethnicity, race, Charlson Comorbidity Index, and body mass index (BMI)

**Table 2:** Mean preventive index scores and ratios for cancer survivors and propensity score matched individuals with no history of cancer

	Cancer survivors *N* = 20,538		Individuals with no history of cancer, *N* = 61,617			
	*N* eligible for screening during study period	Mean (std)	*N* eligible for screening during study period	Mean (std)	Mean difference	Standardized mean difference^a^
Overall Preventive Index Score	20,539	38.4 (24.4)	61,617	34.4 (24.2)	4.0	0.16
Cancer Preventive Index Score	16,588	40.4 (38.7)	50,491	33.2 (36.8)	7.2	0.19
Mammogram	8515	35.8 (37.8)	27,106	29.3 (36.1)	6.5	0.18
Cervical cancer screening	6448	43.9 (42.4)	22,857	38.3 (41.4)	5.6	0.13
Colorectal cancer screening	14,944	39.6 (43.6)	44,637	31.0 (40.6)	8.6	0.21
Cardiovascular Preventive Index Score	20,539	63.7 (33.9)	61,617	59.5 (35.2)	4.2	0.12
Blood pressure screening	20,539	66.8 (37.5)	61,617	61.9 (38.7)	4.9	0.13
HbA1c test	11,583	48.7 (38.7)	34,435	45.0 (38.7)	3.7	0.09
Lipid screening	16,990	72.3 (40.8)	50,536	69.3 (42.4)	3.0	0.07
Abdominal aortic aneurysm screening	2103	15.2 (33.4)	5,909	12.4 (30.3)	2.8	0.09
Infectious Disease Preventive Index Score	20,539	25.4 (29.5)	61,617	22.0 (28.5)	3.4	0.12
Chlamydia screening	145	23.0 (32.7)	419	19.3 (31.0)	3.7	0.12
HIV screening	13,255	8.6 (27.4)	39,783	8.1 (26.7)	0.5	0.02
Flu vaccine	20,539	27.5 (34.6)	61,617	22.7 (32.6)	4.8	0.14
Hepatitis C	11,520	8.3 (26.1)	34,642	6.8 (23.6)	1.5	0.06
Pneumonia vaccine	10,546	47.7 (46.1)	31,557	43.0 (46.1)	4.7	0.10
Mental Health and Substance Abuse Index Score	20,539	23.8 (26.1)	61,617	21.2 (25.0)	2.6	0.10
Depression screening	20,539	21.4 (26.1)	61,617	19.3 (25.1)	2.1	0.08
Substance abuse screening	20,539	26.9 (32.1)	61,617	23.8 (30.9)	2.2	0.10

a The two groups are significantly different when the absolute value of the standardized mean differences is larger than 0.1

Preventive screening indices were calculated as rates where the numerator was the total person-time covered and the denominator was total person-time eligible

**Table 3:** Incidence rate ratios for all cancer survivors compared to propensity score matched individuals with no history of cancer and stratified by cancer type

	All Cancer IRR (95% CI)	Breast IRR (95% CI)	Prostate IRR (95% CI)	Colorectal IRR (95% CI)	Lung IRR (95% CI)
Overall Preventive Index Score	**1.11 (1.09, 1.13)**	**1.22 (1.16, 1.27)**	**1.11 (1.03, 1.17)**	1.05 (0.97, 1.14)	**0.80 (0.71, 0.90)**
Cancer Preventive Index Score	**1.16 (1.12, 1.20)**	**1.21 (1.13, 1.29)**	**1.30 (1.14, 1.49)**	**1.30 (1.15, 1.48)**	0.85 (0.69, 1.04)
Mammogram	**1.18 (1.12, 1.24)**	**1.27 (1.16, 1.40)**	–	1.13 (0.93, 1.39)	0.86 (0.63, 1.16)
Cervical cancer screening	**1.11 (1.05, 1.18)**	**1.15 (1.03, 1.28)**	–	1.18 (0.92, 1.52)	0.88 (0.59, 1.30)
Colorectal cancer screening	**1.22 (1.17, 1.27)**	**1.22 (1.11, 1.34)**	**1.30 (1.14, 1.49)**	**1.39(1.19, 1.63)**	0.85 (0.67, 1.09)
Cardiovascular Preventive Index Score	**1.02 (1.01, 1.03)**	1.01 (0.98, 1.04)	**1.08(1.03, 1.12)**	1.01 (0.97, 1.06)	**0.85 (0.79, 0.92)**
Blood pressure screening	**1.02 (1.00, 1.03)**	1.03 (0.99, 1.06)	**1.07 (1.03, 1.13)**	1.00 (0.95, 1.06)	**0.82 (0.75, 0.89)**
HbA1c test	1.02 (0.99, 1.05)	1.00 (0.94, 1.06)	1.08 (0.98, 1.19)	0.96 (0.87, 1.06)	**0.81 (0.69, 0.95)**
Lipid screening	1.02 (1.00, 1.04)	0.99 (0.95, 1.04)	1.06 (1.00, 1.14)	1.03 (0.95, 1.11)	0.89(0.80, 1.00)
Abdominal aortic aneurysm screening	1.17 (0.92, 1.48)	–	1.49(0.96, 2.33)	1.30 (0.43, 3.93)	0.45(0.10, 2.03)
Infectious Disease Preventive Index Score	**1.07 (1.04, 1.11)**	1.03 (0.95, 1.12)	**1.14 (1.04, 1.25)**	1.01 (0.90, 1.14)	0.90 (0.74, 1.07)
Chlamydia screening	1.06 (0.66, 1.73)	0.26 (0.00, 28.06)	–	–	–
HIV screening	1.06 (0.92, 1.23)	0.86 (0.59, 1.25)	–	–	–
Flu vaccine	**1.13 (1.09, 1.17)**	**1.13 (1.04, 1.23)**	**1.18 (1.06, 1.32)**	1.13 (0.99, 1.30)	0.88(0.72, 1.08)
Hepatitis C	**1.15 (1.01, 1.32)**	0.88 (0.62, 1.25)	0.95 (0.58, 1.54)	0.85 (0.50, 1.43)	–
Pneumonia vaccine	**1.05 (1.01, 1.10)**	1.06 (0.95, 1.17)	1.07 (0.96, 1.20)	1.07 (0.92, 1.26)	1.02 (0.81, 1.30)
Mental Health and Substance Abuse Index Score	1.02 (0.99, 1.05)	1.05 (0.98, 1.12)	1.03 (0.93, 1.14)	1.00 (0.90, 1.11)	**0.75 (0.64, 0.88)**
Depression screening	1.02 (0.98, 1.05)	1.05 (0.98, 1.13)	1.05 (0.94, 1.16)	0.98 (0.88, 1.10)	**0.79 (0.67, 0.94)**
Substance abuse screening	1.02 (0.99, 1.06)	1.04 (0.96, 1.13)	1.01 (0.90, 1.13)	1.02 (0.90, 1.15)	**0.69 (0.57, 0.84)**

*IRR*, incidence rate ratio; *CI*, confidence interval

Bold denotes statistical significance (*p* < 0.05)

Preventive screening indices were calculated as rates where the numerator was the total person-time covered and the denominator was total person-time eligible. All models were adjusted for age, insurance status, smoking status, BMI, race, ethnicity, urban/rural, and number of visits during the study period

## Data Availability

Data available upon reasonable request.
