# Supplementary material for: Preventive service utilization among low-income cancer survivors
Source: J Cancer Surviv. 2021 Aug 19;16(5):1047–54. doi: 10.1007/s11764-021-01095-7 (PMC8857290; doi:10.1007/s11764-021-01095-7)
Supplement: Supplementary file 2 — Supplementary file2 (DOCX 24 kb) [file 11764_2021_1095_MOESM2_ESM.docx]

| **Supplemental Table 2: Demographic characteristics of breast and prostate cancer survivors and the propensity score matched individuals with no history of cancer** | | | | | | |
| --- | --- | --- | --- | --- | --- | --- |
|  | **Breast cancer** | | | **Prostate cancer** | | |
|  | **Cancer survivors** | **Individuals with no history of cancer** | **p-value** | **Cancer survivors** | **Individuals with no history of cancer** | **p-value** |
|  | **N=3,671** | **N=11,013** |  | **N=2,032** | **N=6,096** |  |
| Age |  |  |  |  |  |  |
| 18-19 | 0 | 0 | 0.999 | 0 | 0 | 0.994 |
| 20-34 | 43 (1.2) | 132 (1.2) |  | 1 (0.1) | 3 (0.1) |  |
| 35-49 | 475 (12.9) | 1,433 (13.0) |  | 28 (1.4) | 89 (1.5) |  |
| 50-64 | 1,638 (44.6) | 4,908 (44.6) |  | 643 (31.6) | 1,917 (31.5) |  |
| 65-79 | 1,235 (33.6) | 3,711 (33.7) |  | 1,046 (51.5) | 3,124 (51.3) |  |
| 80+ | 280 (7.6) | 829 (7.5) |  | 314 (15.5) | 963 (15.8) |  |
| Sex |  |  |  |  |  |  |
| Female | 3,671 (100) | 11,013 (100) |  | 0 | 0 |  |
| Male | 0 | 0 |  | 2,032 (100) | 6,096 (100) |  |
| Ethnicity |  |  |  |  |  |  |
| Hispanic | 419 (11.4) | 1,288 (11.7) | 0.103 | 207 (10.2) | 591 (9.7) | 0.716 |
| Non-Hispanic | 3,111 (84.8) | 9,381 (85.2) |  | 1,753 (86.3) | 5,302 (87.0) |  |
| Missing | 141 (3.8) | 344 (3.1) |  | 72 (3.5) | 203 (3.3) |  |
| Race |  |  |  |  |  |  |
| White | 2,982 (81.2) | 9,063 (82.3) | 0.246 | 1,502 (73.9) | 4,558 (74.8) | 0.309 |
| Black or African American | 443 (12.1) | 1,310 (11.9) |  | 405 (19.9) | 1,222 (20.1) |  |
| Asian | 97 (2.6) | 286 (2.6) |  | 37 (1.8) | 97 (1.6) |  |
| American Indian or Alaska Native | 19 (0.5) | 47 (0.4) |  | 15 (0.7) | 37 (0.6) |  |
| Native Hawaiian or other Pacific Islander | 7 (0.2) | 14 (0.1) |  | 6 (0.3) | 5 (0.1) |  |
| Multiple races | 11 (0.3) | 17 (0.2) |  | 11 (0.5) | 33 (0.5) |  |
| Missing | 112 (3.1) | 276 (2.5) |  | 56 (2.8) | 144 (2.4) |  |
| Federal Poverty Level, patient level |  |  |  |  |  |  |
| < 138% | 1,402 (38.2) | 4,666 (42.4) | <0.001 | 608 (29.9) | 1,913 (31.4) | 0.035 |
| 138-200% | 287 (7.8) | 878 (8.0) |  | 117 (5.8) | 445 (7.3) |  |
| > 200% | 325 (8.9) | 998 (9.1) |  | 210 (10.3) | 585 (9.6) |  |
| Missing | 1,657 (45.1) | 4,471 (40.6) |  | 1,097 (54.0) | 3,153 (51.7) |  |
| Insurance status at most recent visit |  |  |  |  |  |  |
| Private | 593 (16.2) | 1,756 (15.9) | <0.001 | 287 (14.1) | 698 (11.5) | <0.001 |
| Medicaid | 778 (21.2) | 1,766 (16.0) |  | 182 (9.0) | 672 (11.0) |  |
| Medicare | 1,545 (42.1) | 4,243 (28.5) |  | 1,248 (61.4) | 3,488 (57.2) |  |
| Uninsured | 655 (17.8) | 2,911 (26.4) |  | 277 (13.6) | 1,136 (18.6) |  |
| Other/unknown | 100 (2.7) | 337 (3.1) |  | 38 (1.9) | 102 (1.7) |  |
| Patient region of residence |  |  |  |  |  |  |
| West | 2,928 (79.8) | 8,621 (78.3) | <0.001 | 1,504 (74.0) | 4,476 (73.4) | 0.034 |
| Southwest | 22 (0.6) | 98 (0.9) |  | 10 (0.5) | 33 (0.5) |  |
| Midwest | 363 (9.9) | 1,434 (13.0) |  | 256 (12.6) | 923 (15.1) |  |
| Southeast | 326 (8.9) | 778 (7.1) |  | 237 (11.7) | 608 (10.0) |  |
| Northeast | 15 (0.4) | 47 (0.4) |  | 10 (0.5) | 28 (0.5) |  |
| Missing | 17 (0.5) | 35 (0.5) |  | 15 (0.8) | 28 (0.45) |  |
| BMI |  |  |  |  |  |  |
| Underweight (<18.5 kg/m^2^) | 58 (1.6) | 153 (1.4) | 0.917 | 19 (0.9) | 41 (0.7) | 0.762 |
| Normal (18.5-24.9 kg/m^2^) | 895 (24.4) | 2,698 (24.5) |  | 407 (20.0) | 1,216 (20.0) |  |
| Overweight (25.0-29.9 kg/m^2^) | 981 (26.7) | 2,934 (26.6) |  | 798 (39.3) | 2,384 (39.1) |  |
| Obese (≥ 30 kg/m^2^) | 1,454 (39.6) | 4,401 (40.0) |  | 656 (32.3) | 2,015 (33.1) |  |
| Missing | 283 (7.7) | 827 (7.5) |  | 152 (7.5) | 440 (7.2) |  |
| Smoking Status |  |  |  |  |  |  |
| Current smoker | 659 (18.0) | 2,168 (19.7) | 0.023 | 314 (15.5) | 1,183 (19.4) | <0.001 |
| Former smoker | 935 (25.5) | 2,614 (23.7) |  | 834 (41.0) | 23,07 (37.8) |  |
| Never smoker | 1,905 (51.9) | 5,655 (51.4) |  | 791 (38.9) | 2,290 (37.6) |  |
| Missing | 172 (4.7) | 576 (5.2) |  | 93 (4.6) | 316 (5.2) |  |
| Rurality |  |  |  |  |  |  |
| Rural | 1,273 (34.7) | 3,789 (34.4) | 0.488 | 782 (38.5) | 2,341 (38.4) | 0.321 |
| Urban | 2,382 (64.9) | 7,190 (65.3) |  | 1,235 (60.8) | 3,727 (61.1) |  |
| Missing | 16 (0.4) | 34 (0.3) |  | 15 (0.7) | 28 (0.5) |  |
| Charlson Comorbidity Index |  |  |  |  |  |  |
| 0 | 1,035 (28.2) | 3,119 (28.3) | 0.977 | 458 (22.5) | 1,421 (23.3) | 0.775 |
| 1 | 909 (24.8) | 2,709 (24.6) |  | 527 (25.9) | 1,566 (25.7) |  |
| 2+ | 1,727 (47.0) | 5,185 (47.1) |  | 1,047 (51.5) | 3,109 (51.0) |  |
| Number of visits between 2014-2017 |  |  |  |  |  |  |
| 0 | 806 (22.0) | 2,860 (26.0) | <0.001 | 410 (20.2) | 1,669 (27.4) | <0.001 |
| 1-4 | 530 (14.4) | 1,790 (16.3) |  | 310 (15.3) | 973 (16.0) |  |
| 5-12 | 939 (25.6) | 2,722 (24.7) |  | 532 (26.2) | 1,555 (25.5) |  |
| 13+ | 1,396 (38.0) | 3,641 (33.1) |  | 780 (38.4) | 1,899 (31.2) |  |

Cancer survivors were included if they were diagnosed with malignant cancer before the start of the study period. Cancer survivors were identified through diagnoses codes and problem lists within their medical records. Each cancer survivor was propensity score matched to three patients with no history of cancer on age at the beginning of the study period, urban/rural, ethnicity, race, Charlson Comorbidity Index, and body mass index (BMI).
