# Supplementary material for: Preventive service utilization among low-income cancer survivors
Source: J Cancer Surviv. Author manuscript; Available in PMC 2022 Oct 1. (PMC8857290; doi:10.1007/s11764-021-01095-7)
Supplement: Supplementary file 1 [file NIHMS1752361-supplement-Supplemental_Table_1.docx]

**Supplemental Table 1. Definitions of Services Examined, Inclusion and Exclusion Criteria, and Interval After Which a Service is Considered “Due”**

| Servicea | Inclusion criteria | Exclusion criteria | Interval | Test |
| --- | --- | --- | --- | --- |
| Colorectal cancer screening | Age 50-75 years | None | 1 year (occult) 5 years (sigmoid) 10 years | Fecal occult Sigmoidoscopy Colonoscopy |
| Breast cancer screening | Female and age 50-74 years | History of bilateral mastectomy | 2 years | Mammography |
| Cervical cancer screening | Female and age 21-65 years | History of hysterectomy | 3 years (cytology) 5 years (age 30-65 plus co-testing with HPV) | Pap Or Pap plus HPV |
| Chlamydia screening | Female, age <25 years, and indication of sexual activity^b^ | None | Annually | Urine GC/CT GC/CT swab GC/CT culture |
| HIV screening | Age 19-65 years | Prior diagnosis of HIV | Once | HIV Ab, Rapid HIV, HIV RNA |
| Influenza vaccine | Age ≥19 years | None | Annually during flu season 8/1-7/31^d^ | Flu, flumist, high-dose flu |
| Pneumonia vaccine | Age ≥65 years | None | Once |  |
| Hepatitis C screening | Date of birth 1945-1965 | Prior diagnosis of hepatitis C | Once | Hepatitis C Ab |
| Lipid screening | Female: age ≥45 years, and increased risk (diabetes, CAD, PVD, AA carotid stenosis, tobacco, HTN, obesity) Male: age ≥35 years | None | 5 years | Lipid panel Direct LDL |
| Diabetes screening | Ages 40-70 years, overweight | None | Annually | HbA1c, Fasting glucose, Glucose tolerance test |
| Blood pressure screening | Age ≥19 years | None | 5 years (age 19-39) Annually (age ≥40 years) | Blood pressure |
| AAA screening | Male, age ≥65 years, and current or ever smoker | None | Once | Aortic ultrasound |
| Depression screening | Age ≥19 years | None | Annually | PHQ2 |
| Substance abuse screening^d^ | Age ≥19 years | None | Annually | SBIRT |
| Aggregate preventive index^e^ | Age ≥19 years | None | N/A | All of the above except AAA ultrasound^f^ |

^a^ Patients were considered “covered” for preventive services when services were documented as received as opposed to ordered only.

^b^ Sexual activity was inferred from procedures, diagnoses, lab tests, and medications considered in meaningful use reporting to indicate sexually active women, and include history of pregnancy, sexually transmitted disease, and contraceptive devices and medications.

^c^ Overweight identified as BMI >25, weight >220, or active diagnosis of obesity.

^d^ Substance abuse screening is captured for the 2015 calendar year only because 2014 was the first full year for which substance screening history was available in OCHIN’s electronic health record.

^e^ Aggregate preventive index does not include AAA screening due to limited eligible patients.

AAA, abdominal aortic aneurysm; Ab, antibody; CAD, coronary artery disease; GC/CT, gonorrhea/chlamydia; HPV, human papillomavirus; HTN, hypertension; LDL, low-density lipoprotein; PVD, peripheral vascular disease; RNA, ribonucleic acid; PHQ2, Patient Health Questionnaire-2; SBIRT, Screening, Brief Intervention, and Referral to Treatment; N/A, not applicable.

Source: Hatch BA, Tillotson CJ, Huguet N, Hoopes MJ, Marino M, DeVoe JE. Use of a Preventive Index to Examine Clinic-Level Factors Associated With Delivery of Preventive Care. *Am J Prev Med.* 2019;57(2):241-249.
